# Circulating Leukocytes and Antibody Isotype Reactivity in Rats Experimentally Infected with Different Burdens of *Strongyloides venezuelensis*, Before and After Treatment with Ivermectin or Dexamethasone

**DOI:** 10.3390/tropicalmed11090242

**Published:** 2026-08-26

**Authors:** João Gustavo Mendes Rodrigues, Guilherme Silva Miranda, Genil Mororó Araújo Camelo, Caio Brandão Goes Gouveia, Deborah Aparecida Negrão-Corrêa

**Affiliations:** Department of Parasitology, Institute of Biological Sciences, Federal University of Minas Gerais, Belo Horizonte 31270-901, Brazil; gustavorodrigues_98@hotmail.com (J.G.M.R.); guilherme.miranda@ifma.edu.br (G.S.M.); genilmororo@gmail.com (G.M.A.C.); caiobggouveia@gmail.com (C.B.G.G.)

**Keywords:** *Strongyloides*, immunological diagnosis, isotype reactivity to parasite antigens

## Abstract

Strongyloidiasis is a neglected, potentially lifelong disease caused by *Strongyloides stercoralis* that can lead to a high mortality rate in immunocompromised hosts; therefore, it is necessary to increase the accuracy of infection diagnosis. In the current study, we used Wistar rats experimentally infected with different burdens of *Strongyloides venezuelensis* to characterize circulating leukocytes and IgM, IgG, IgG1, IgG2a, and IgA reactivity before and after anthelmintic treatment or immunosuppression. Infection, especially with 500 L3, induced an increase in circulating leukocytes, particularly during the acute phase. Animals treated with ivermectin showed complete elimination of the parasite and an early reduction in circulating eosinophils. The production of IgM, IgG, and IgG1 anti-L3 (infective larvae) and anti-Sv (adult worm) antigens was significantly elevated in all infected groups but did not allow differentiation between cured and infected animals. Treatment with dexamethasone delayed worm elimination and temporarily reduced cellular response and parasite-specific IgG production but did not alter IgM reactivity. IgG2a anti-L3 and anti-Sv reactivity showed a progressive reduction, returning to baseline levels in ivermectin-treated rats. IgA anti-ES/L3 (excretory and secretory larval antigens) reactivity significantly decreased in intestinal wash after parasitological cure. These data would help the development of more efficient immunodiagnostic alternatives for human strongyloidiasis based on antibody isotype reactivity.

## 1. Introduction

Strongyloidiasis is a neglected tropical disease caused mainly by *Strongyloides stercoralis*, which is estimated to affect between 300 and 600 million people, especially in tropical and humid regions [1,2]. In most cases, infection with *S. stercoralis* results in an asymptomatic chronic disease that can persist lifelong in the human host due to the parasite’s autoinfective capacity [3,4]. In immunocompetent individuals, the parasite burden remains very low, leading to low larval output in feces, which makes the diagnosis and control of strongyloidiasis challenging. However, strongyloidiasis can progress to the life-threatening hyperinfection syndrome, a fatal condition in up to 85% of cases, in immunocompromised individuals [3,5,6]. Therefore, accurate diagnosis and early treatment of strongyloidiasis are essential to eliminate the risk of a future hyperinfection [7,8,9].

The definitive diagnosis of human strongyloidiasis is based on the identification of parasite larvae in fecal samples; however, due to the low and intermittent production of larvae by the parthenogenetic female of *S. stercoralis* [10,11], routine fecal examination methods lack sensitivity. Parasitological methods based on larval concentration, such as the agar plate culture, Baermann–Moraes, or Rugai techniques, have shown increased sensitivity for the diagnosis of strongyloidiasis; however, these methods are time-consuming, require trained parasitologists, and, unless multiple stool samples are tested, remain incapable of identifying all infected individuals [9,12,13]. Therefore, immunological and/or molecular diagnostic tests have been suggested to overcome the low sensitivity of parasitological stool examination [14,15,16].

At present, the sensitivity and specificity of different molecular techniques evaluated for the diagnosis of strongyloidiasis show considerable variability and require further validation studies before being used as diagnostic tools in exposed populations [16,17]. For this reason, serological assays, such as the enzyme-linked immunosorbent assay (ELISA), have been proposed as screening alternatives to detect *S. stercoralis* infection [6,15,16,18]. Serological assays are easy to perform, allow the simultaneous testing of large numbers of samples, and have shown greater sensitivity for the diagnosis of strongyloidiasis, particularly in non-endemic areas [9,10,14,19]. However, there is still a lack of standardization and validation of different serological assays applied to the diagnosis of strongyloidiasis [18]. Moreover, serological assays require antigen production and may present cross-reactivity with other helminth infections, lower sensitivity in samples from immunosuppressed hosts, and an inability to differentiate between current and past infection [18,20,21]. These limitations should be addressed to improve serological methods for the diagnosis of strongyloidiasis, especially in endemic areas where the population is exposed to infection with different nematode species. There is evidence that the measurement of different antibody isotypes may help improve the sensitivity and specificity of serological assays for the diagnosis of chronic strongyloidiasis [22,23,24]; however, this topic requires further investigation. Moreover, aspects such as the time of infection, parasite load, and confirmation of parasitological cure after anthelmintic treatment are difficult to investigate in human populations, and experimental infection models may be particularly useful.

In this context, *S. venezuelensis* presents itself as a useful model. It is a species that naturally parasitizes rodents, migrates through the host lungs, and establishes in the small intestinal mucosa, similarly to the development of *S. stercoralis* in humans [25,26]. Moreover, *S. venezuelensis* and *S. stercoralis* share high genomic, proteomic, and antigenic sequence homology [5], and published data have shown that heterologous antigens derived from *S. venezuelensis* have good sensitivity and specificity for the diagnosis of human strongyloidiasis [27,28,29]. It is worth highlighting that the majority of experimental studies investigating different aspects of strongyloidiasis use *S. venezuelensis* infection in mice; however, in this experimental model, strongyloidiasis is self-limiting after 10–14 days [30]. Therefore, the mouse model of infection is not appropriated for evaluating the humoral response. In contrast, infection with *S. venezuelensis* lasts for a longer period in rats and gerbils [31], and the experimental rat model has been used to evaluate diagnostic alternatives for strongyloidiasis [32,33].

Therefore, in the present experimental study, we infected Wistar rats with *S. venezuelensis* to evaluate the effects of parasite burden and anthelmintic and immunosuppressive treatments on the reactivity of different antibody isotypes to various parasite antigens. Our data show that plasma anti-L3 IgG2a reactivity and anti-L3/Excretory–secretory (ES) intestinal IgA reactivity perform better at detecting active infection. In addition, IgM has the potential to detect infection even during immunosuppressive regimens.

## 2. Material and Methods

### 2.1. Animals and Infection with S. venezuelensis

Male Wistar rats aged between 4 and 6 weeks (~120 g), specific-pathogen-free (SPF), were obtained from the Animal Facility of the Federal University of Minas Gerais (UFMG, Belo Horizonte, Brazil). During the experimental procedures, the rats were housed in the Animal Facility of the Department of Parasitology (UFMG, Brazil) designated for infected animals, fed standard laboratory chow (Presence, São Paulo, Brazil), and provided water ad libitum. All animal care and experimental procedures were conducted in accordance with the ARRIVE guidelines and were previously approved by the Ethics Committee on the Use of Animals (CEUA/UFMG) under protocol number 129/2020.

For experimental infections, infective third-stage filariform larvae (L3) of *S. venezuelensis* were obtained from stool cultures of infected rats and recovered using the Baermann–Moraes technique [34,35]. The larvae were then filtered, concentrated, washed (3–4 times) in phosphate buffer (PBS—13.7 mM NaCl, 0.27 mM KCl, 0.14 mM KH_2_PO_4_, and 0.43 mM Na_2_HPO_4_·7H_2_O), quantified under a stereomicroscope, and used for experimental infections [36,37]. The rats were inoculated subcutaneously in the abdominal region with 100 μL of phosphate-buffered saline (PBS) containing 10 (low burden) or 500 (high burden) infective larvae per rat, according to Schilter et al. [38].

### 2.2. Experimental Protocol

**Figure 1:**
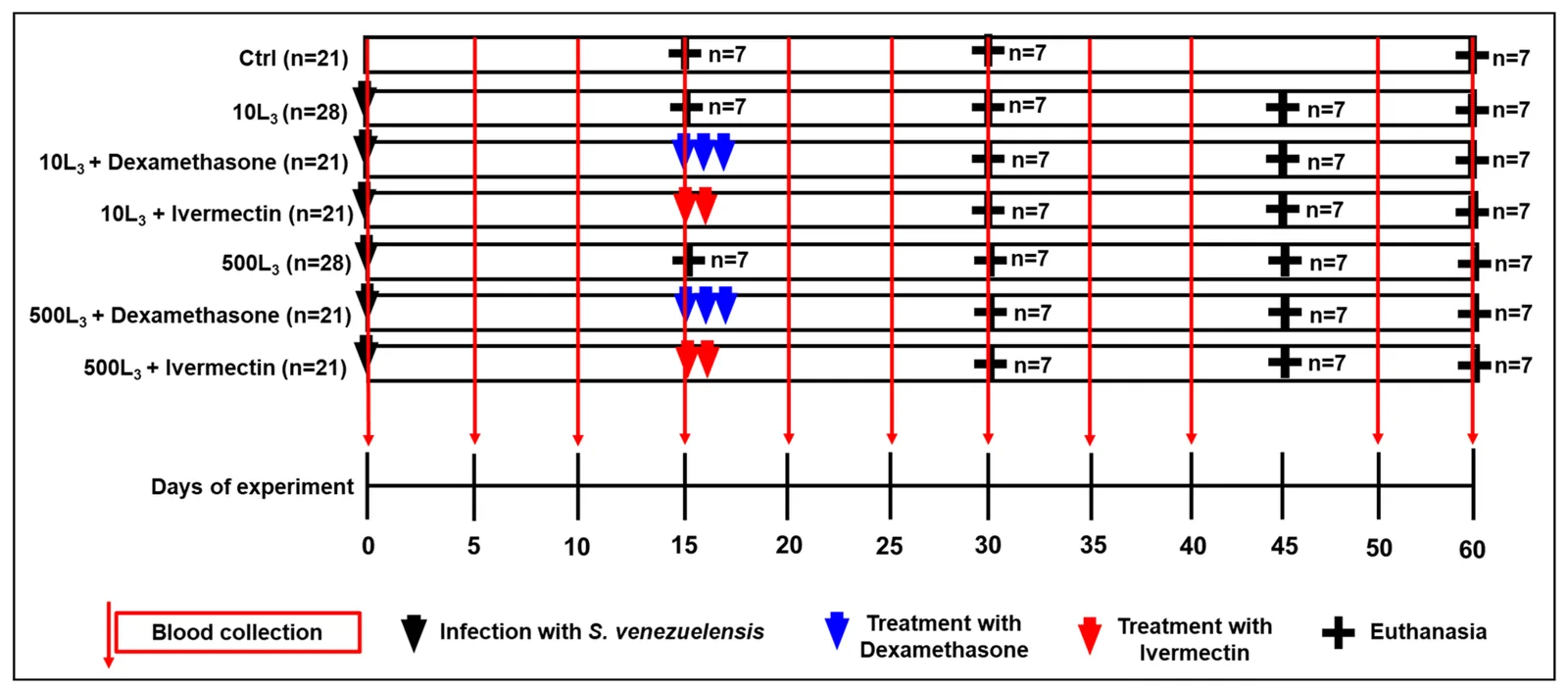
Experimental design. Male Wistar rats were infected subcutaneously with 10 (low burden) or 500 L3 larvae (high burden) of *S. venezuelensis* on day 0. On day 15, infected rats from the Sv + ivermectin group received a dose of 200 μg/Kg/day of ivermectin orally for 2 consecutive days, as indicated by the blue arrow head (Groups 10 L3 + ivermectin and 500 L3 + ivermectin), and infected rats from the Sv + dexamethasone group received a dose of 4 mg/Kg/day of Injectable Decadron intraperitoneally for 3 consecutive days, as indicated by the blue arrow head (Groups 10 L3 + dexamethasone and 500 L3 + dexamethasone). Another infected group was maintained without treatment (Groups 10 L3 and 500 L3). The blood was sampled at 0, 5, 10, 15, 20, 25, 30, 35, 40, 50, and 60 days of the experimental procedure, indicated as thin red arrows an labeled as blood collection, for leukometric analysis and antibody reactivity in plasma. Parasitological evaluation and intestinal lavage collection were conducted at defined points following euthanasia. Euthanasia (**+**) of 7 (seven) rats/group was performed at 15, 30, 45, and 60 days of the study, as indicated.

For each experimental procedure (low or high parasite burden), the rats were randomly assigned to four experimental groups: a control group (Ctrl) composed of uninfected and untreated animals; an infected group (Sv) composed of rats infected with *S. venezuelensis* that did not receive any treatment; an infected and immunosuppressed group (Sv + dexamethasone) composed of rats infected with *S. venezuelensis* and treated with the immunosuppressant dexamethasone; and an infected and cured group (Sv + ivermectin) composed of rats infected with *S. venezuelensis* and treated with the anthelmintic ivermectin (Figure 1). Parasite infection was performed on day 0 (zero) for all infected groups. Immunosuppressive treatment was performed on days 15, 16, and 17 post-infection with dexamethasone disodium phosphate (Decadron Injetável, Aché Laboratórios Farmacêuticos S.A., São Paulo, Brazil) solution (4 mg/Kg/day) administered via the intraperitoneal (IP) route [39,40]. Anthelmintic treatment was performed on days 15 and 16 post-infection with ivermectin (Ivermec 1%—Chemitac^®^, Ipiranga, São Paulo, Brazil) administered orally at a dose of 200 μg/Kg/day [41]. The experimental procedures evaluated the effects of two infective doses of *S. venezuelensis*: for the high-burden infection, each rat was inoculated with 500 infective larvae (L3), whereas for the low-burden infection, each rat received 10 L3 (Figure 1).

Blood samples were collected from the lateral tail vein of seven animals per group at days 0, 5, 10, 15, 20, 25, 30, 35, 40, 50, and 60 of the experimental protocol for leukometric evaluation and immunoenzymatic assays (Figure 1). For parasitological assessment, seven other animals from the Sv experimental group were euthanized at 15, 30, and 45 days post-infection (dpi), while from Sv + dexamethasone and Sv + ivermectin, seven other rats were euthanized at 30 and 45 dpi, since all of the infected groups had been exposed to the same procedures up to 15 dpi (Figure 1). At 60 dpi, the animals used for the immunological evaluation in each experimental group were also euthanized to confirm parasitological cure. Infected animals euthanized at 15, 30, and 60 dpi were additionally used to assess intestinal washes. At these same time points, seven animals from the control group (uninfected and untreated) were euthanized for intestinal wash collection and immunological assessment (Figure 1). The leukocyte and parasitological analyses of the animal samples were performed in a blinded manner.

For euthanasia, the animals received an overdose of ketamine (240 mg/Kg, Cetamin^®^, Syntec, Santana do Parnaíba, São Paulo, Brazil) combined with xylazine hydrochloride 45 mg/Kg, Dopaser^®^, HertapeCalier, Embu-Guaçu, São Paulo, Brazil) via IP injection, followed by cervical dislocation. After euthanasia, the anterior half (duodenum and proximal jejunum) of the small intestine of these animals was surgically removed and immediately washed with PBS to obtain the intestinal lavage, and the mucosa was used for the recovery and counting of adult worms (Figure 1).

### 2.3. Blood and Plasma Collection and Leukometric Assessment

For blood collection, seven rats from each experimental group were immobilized in a restraining holder at 0, 5, 10, 15, 20, 25, 30, 35, 40, 50, and 60 days of the experimental procedure, and 0.2–0.4 mL of blood was collected directly from the tail vein and placed in tubes containing EDTA (Microtube FL5-1105, FirstLab, Paraná, Brazil), as described by Zou et al. [42]. Next, 30 µL of each blood sample was used for total and differential leukocyte counts. For this purpose, a 10 µL sample of whole blood was used to prepare a blood smear, which was fixed, and the cells were differentially stained using the rapid panoptic kit (Renylab^®^, Minas Gerais, Brazil). The remaining 20 µL of whole blood was diluted in Turck’s solution, and the total number of leukocytes was estimated using a Neubauer chamber. One hundred leukocytes from each smear were evaluated and classified as lymphocytes, monocytes, neutrophils, or eosinophils based on morphological characteristics [43]. The remainder of the blood sample was centrifuged (3000× *g*, 4 °C for 10 min), and the plasma contained in the supernatant was individually collected and stored at −20 °C for subsequent ELISA.

### 2.4. Intestinal Lavage and Worm and Larval Recovery

The intestinal lavage was obtained by flushing the proximal half of the small intestine (duodenum and proximal jejunum) of each experimental animal with 3 mL of cold sterile PBS containing a protease inhibitor cocktail (one tablet in 25 mL of PBS; Boehringer Mannheim, Indianapolis, IN, USA). The recovered solution was centrifuged (3000× *g* at 4 °C for 10 min), and the supernatant was stored at −20 °C and used to evaluate IgA reactivity to parasite antigens.

Adult worms of *S. venezuelensis* were recovered after collection of the intestinal lavage. The anterior half of the small intestine was sectioned longitudinally, placed on a mesh inside a sedimentation cup containing saline solution (0.85% NaCl), and incubated in a water bath at 37 °C for 4 h. Subsequently, the adult worms deposited at the bottom of the sedimentation cup were transferred to Petri dishes containing PBS and quantified under a stereoscopic microscope. The intestinal wall was also examined to quantify worms that were still attached to the mucosa, and the total worm count was expressed as the number of worms per small intestine [36]. Fecal samples from the same infected animals were collected, weighed, and processed using the modified Rugai parasitological method, as described by Rodrigues et al. [44]. The recovered *S. venezuelensis* larvae were quantified and expressed as the number of larvae per gram of feces.

### 2.5. S. venezuelensis Antigens

Filariform larvae (L3) and adult worms (Sv) of *S. venezuelensis* were used to obtain crude extracts, according to a method previously described by Fernandes et al. [45]. Briefly, the recovered larvae and worms were decontaminated using a 0.25% sodium hypochlorite solution followed by incubation in an antibiotic cocktail solution (4 mg/mL of Gentamicin, 1000 U/mL of Penicillin and 1000 μg/mL of Streptomycin), as described by Barçante et al. [46]. After several washes, the parasites were resuspended in PBS (1 mL of L3 or worm pellet/3 mL of PBS) containing a protease inhibitor cocktail (1 tablet in 25 mL of PBS; Boehringer Mannheim). The parasite suspensions were maintained in an ice bath and processed in a tissue homogenizer (Power General 125, Fisher Scientific, Pittsburgh, PA, USA) for 10 cycles of 30 s with 1 min intervals. Glass beads (212–300 microns, Sigma Chemical Co. St. Louis, MI, USA) were then added to the suspensions, which were vortexed for 15 min. Subsequently, the parasite suspensions were recovered and sonicated using an ultrasonic processor (PGC Scientific, Gaithersburg, MD, USA) for 5 cycles of 30 s at high power with 1 min intervals. The homogenates were centrifuged at 3000× *g* for 1 h and the soluble proteins were recovered from the supernatant, aliquoted, and stored at −20 °C until use.

ES antigens from L3 larvae and adult worms of *S. venezuelensis* were prepared according to the method of Cançado et al. [47]. Briefly, after the decontamination described above, the parasites were cultured for 24 h in RPMI 1640 medium containing 100 U/mL penicillin, 100 μg/mL streptomycin, and 0.25 μg/mL amphotericin B (Sigma-Aldrich, St. Louis, MI, USA) at 37 °C with 5% CO_2_ in a humidified incubator (CO_2_ Water Jacketed Incubator, Harris Manufacturing Company, North Billerica, MA, USA). During the culture period for excretory–secretory (E/S) antigen collection, parasite viability (larvae and adult worms) was continuously verified by visual observation of active motility under an inverted microscope. The ES products present in the culture supernatant were collected, centrifuged (210× *g* for 3 min), and concentrated using centrifugal filter units with a 10 kDa cut-off membrane (Centricon, Millipore, Bedford, MA, USA). The concentrated supernatants were collected and stored at −20 °C until use.

The protein concentration of the *S. venezuelensis* antigens was determined using the Pierce BCA protein assay according to the manufacturer’s instructions (PierceTM BCA Protein Assay Kit, Thermo Fisher Scientific, Waltham, MA, USA). To eliminate batch-to-batch variation, a single lot of each antigen preparation was produced, aliquoted, and used across all experimental assays in this study.

### 2.6. Antibody Detection by Means of Immunoenzymatic Assay (ELISA)

The reactivity of IgM, IgA, total IgG, and the IgG1 and IgG2a isotypes to parasite antigenic extracts was evaluated by means of ELISA in plasma samples collected from each experimental animal. Initially, the immunoenzymatic assays were optimized using different dilutions of each reagent. For the assays, 96-well plates (Costar^®^ 96 Well EIA/RIA Assay Microplate, Corning Incorporated, Corning, NY, USA) were coated for 12 h at 4 °C with 100 µL per well of carbonate-bicarbonate buffer (0.5 M Na_2_CO_3_ and 0.5 M NaHCO_3_, pH 9.6) containing 5 µg/mL of soluble larval (L3) or adult worm (Sv) antigen, or 10 µg/mL of excretory–secretory antigen from L3 larvae (ES/L3) or adult worms (ES/Sv).

After this period and between each incubation step, the plates were washed four times with washing buffer (PBS containing 0.05% Tween 20—Sigma-Aldrich, San Luis, MO, USA). Subsequently, the plates were blocked for 1 h at room temperature with washing buffer containing 1% bovine serum albumin (BSA—Sigma-Aldrich) or 2% skimmed milk (Molico^®^, Nestlé, São Paulo, SP, Brazil), as specified in Table 1. After blocking, plasma samples from each experimental animal, diluted 1:200 in dilution buffer (washing buffer containing 0.1% BSA or 0.2% skimmed milk), were added to the plates in duplicate and incubated for 2 h at room temperature. Next, 100 µL per well of dilution buffer containing anti-rat IgG1 or IgG2a antibodies directly conjugated to peroxidase was added, and the plates were incubated for 2 h at room temperature. For the IgG, IgM, and IgA assays, dilution buffer containing biotin-conjugated antibodies was added and incubated for 2 h, followed by a 1 h incubation with 100 μL per well of blocking buffer containing peroxidase-conjugated streptavidin (Streptavidin-HRP B, 2 mL BioTechne, R&D Systems, Minneapolis, MN, USA) (Table 1).

The reaction was developed by adding 100 μL per well of citrate buffer (0.1 M C_6_H_8_O_7_·H_2_O, 0.2 M Na_2_HPO_4_, pH 5) containing o-phenylenediamine dihydrochloride (Thermo Scientific, USA) and 0.05 M hydrogen peroxide substrate, and after 30 min, the reaction was stopped with 50 µL per well of 2 N sulfuric acid. The absorbance of the samples was measured at 492 nm using a microplate reader (VersaMax, Molecular Devices, San Jose, CA, USA).

Plasma samples collected on day 0 from negative (*n* = 7) and positive control animals (*n* = 7 per group, low or high burden) were added to each plate. Data were presented as relative reactivity (RR), calculated by dividing the absorbance of each sample by the cut-off value. The cut-off was defined as the mean absorbance plus two standard deviations of the same uninfected baseline group (*n* = 7, day 0), which was consistently used across all plates. Therefore, samples with RR ≤ 1 were considered non-reactive, whereas samples with RR > 1 were considered reactive to the parasite antigen.

The ELISA applied to assess IgA reactivity to parasite antigens in intestinal lavage samples followed a methodological sequence similar to that described for IgA reactivity in plasma. However, the plates were coated with 10 µg/mL of L3 or adult worm antigen, or 20 µg/mL of ES antigen from L3 larvae or adult worms. The intestinal lavage samples were diluted 1:4 in dilution buffer, and the anti-IgA antibody was diluted 1:10,000.

### 2.7. Statistical Analyses

The sample size of the experimental protocol was determined using the formula proposed by Sampaio [48] at a 95% confidence level. To estimate the variation across the most variable parameters (parasitological, immunological, and hematological data), published data on similar subjects were used.

All data were initially analyzed for normality using the Shapiro–Wilk test. Data collected longitudinally from the same animals, such as antibody reactivity and circulating leukocytes, were analyzed using a two-way mixed-effects model with the Geisser–Greenhouse correction. Post hoc multiple comparisons between groups at specific time points were performed using Tukey’s test. For comparisons of count data between groups, the Kruskal–Wallis test followed by Dunn’s post hoc correction were employed. Frequencies were expressed as percentages (%) and compared using Fisher’s exact test. Values of *p* < 0.05 were considered statistically significant. GraphPad Prism (version 8, Prism Software, Irvine, CA, USA) was used to perform the statistical analyses and generate the graphs.

## 3. Results

### 3.1. Parasite Burden and Worm Elimination

Adult worms of *S. venezuelensis* were recovered from the small intestine, and parasite larvae were recovered from the feces of all infected rats at 15 dpi, independently of the infective dose. In rats infected with 10 L3 per rat, an average of 3.4 ± 0.4 worms were recovered from the intestine and 615.1 ± 107.6 larvae/g of feces were detected, whereas in rats infected with 500 L3, 78.1 ± 7.7 worms were recovered from the small intestine and 3806.1 ± 553.3 larvae/g of feces were detected (Table 2). At 30 dpi, a significant reduction in the number of worms recovered from the intestine and larvae detected in feces was observed in untreated rats, and after 45 dpi, no intestinal worms or fecal larvae were recovered from animals in either the high- or low-parasite burden groups, indicating complete elimination of the infection (Table 2).

Dexamethasone treatment resulted in a delay in worm elimination in rats infected with a high parasite burden, which showed significantly higher mean numbers of worms (57.3 ± 5.6 vs. 36.3 ± 2.8) and larvae (824.5 ± 529.5 vs. 356.2 ± 176.0) at 30 dpi than untreated infected rats. At the same time point, rats infected with a low parasite burden showed no significant difference in the number of worms recovered from the intestine between the untreated infected group and the infected group treated with dexamethasone; however, the number of larvae eliminated in the feces was significantly higher in low-burden infected rats that received dexamethasone treatment compared with untreated infected animals (451.9 ± 97.3 vs. 254.0 ± 132.4, respectively). Similarly to that observed in high-burden infected rats, no worms or larvae were detected at 45 dpi in rats infected with 10 L3 per rat across the different experimental groups (Table 2). All infected rats that received ivermectin treatment at 15 dpi showed no worms in the small intestine or larvae in the feces at 30 dpi, regardless of the infective dose, demonstrating that the treatment was effective in achieving parasitological cure (Table 2).

### 3.2. Total and Differential Leukocyte Count

**Figure 2:**
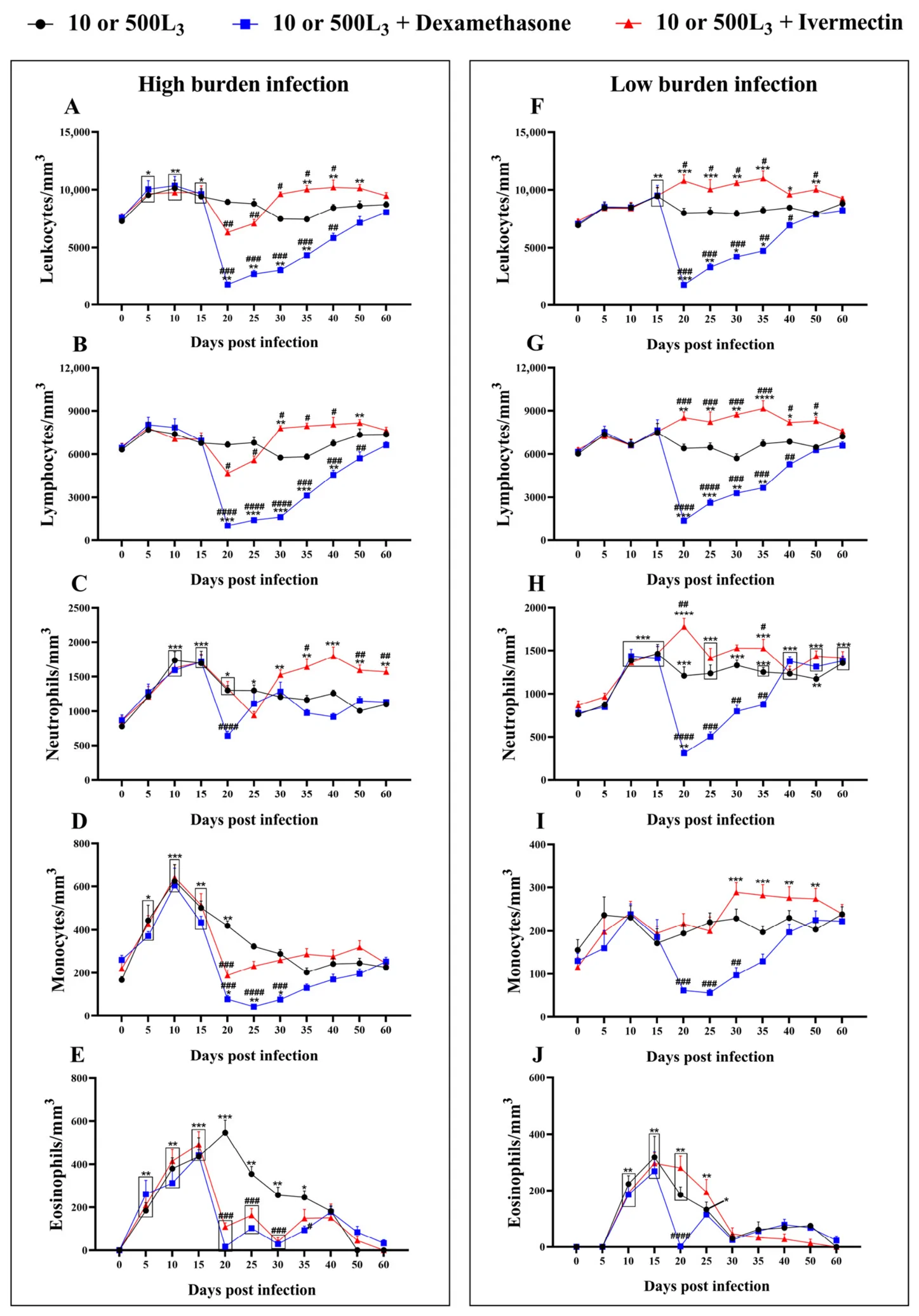
Total and differential leukocyte counts in the peripheral blood of Wistar rats infected with 500 L3 or 10 L3 of *S. venezuelensis*, treated or not treated with dexamethasone or ivermectin. Number of total leukocytes (**A**,**F**), lymphocytes (**B**,**G**), neutrophils (**C**,**H**), monocytes (**D**,**I**), and eosinophils (**E**,**J**). (**A**–**E**): Rats were infected with 500 L3 per rat (high parasite burden). (**F**–**J**): Rats infected with 10 L3 per rat (low parasite burden). At 0 (before infection), 5, 10, 15, 20, 25, 30, 35, 40, 50, and 60 days after infection, the blood samples were collected from the lateral tail veins for leukocyte count. 10 L3 or 500 L3 = infected-only animals; 10 L3 + dexamethasone or 500 L3 + dexamethasone = animals infected and treated via IP with 4 mg/Kg/day of Decadron at 15 dpi for 3 days; 10 L3 + ivermectin or 500 L3 + ivermectin = rats infected and treated orally with 200 μg/Kg/day of ivermectin at 15 dpi for 2 consecutive days. Data are plotted as mean ± standard deviation (SD) and were analyzed by means of a two-way mixed-effects model with Geisser–Greenhouse correction, followed by Tukey’s post hoc test. *n* = 7 rats/group. * represents a statistically significant difference in comparison to day 0 or the non-infected control; # represents a statistically significant difference in comparison to the infected-only group at the same time point. # or * *p* < 0.05; ## or ** *p* < 0.01; ### or *** *p* < 0.001; #### or **** *p* < 0.0001. The boxes highlight diffrent experimental groups that share statistic significance in comparison to unfected (*) or to infected control (#).

Infection with *S. venezuelensis* altered the profile of circulating leukocytes in the different experimental groups (Figure 2). Infection of rats with 500 L3 per rat induced a significant increase in the total number of circulating leukocytes between 5 and 15 dpi, and leukocyte counts returned to baseline levels after 20 dpi (Figure 2A). The increase in circulating leukocytes was accompanied by significantly higher numbers of neutrophils (Figure 2C), monocytes (Figure 2D), and eosinophils (Figure 2E), but not lymphocytes (Figure 2B). Circulating eosinophils remained significantly elevated from 5 to 40 dpi, coinciding with the presence of the parasite in the host (Table 2). In contrast, rats infected with a low parasite burden (10 L3 per rat) showed small variations in the number of circulating leukocytes, with a significant increase detected only at 15 dpi (Figure 2F) and no significant changes observed in lymphocyte (Figure 2G) and monocyte counts (Figure 2I). However, the number of circulating neutrophils in these rats remained significantly elevated between 10 and 60 dpi (Figure 2H), whereas eosinophil counts remained significantly elevated between 10 and 25 dpi (Figure 2J).

Rats infected and treated with dexamethasone (Sv + dexamethasone) showed a marked and transient reduction in the number of leukocytes between days 20 and 35 post-infection compared with uninfected and other infected groups, independent of parasite burden (Figure 2A,F). The reduction in circulating leukocytes was accompanied by a significant decrease in the numbers of lymphocytes (Figure 2B,G), monocytes (Figure 2D,I), and eosinophils (Figure 2E,J), whereas a significant reduction in neutrophils was observed only at 20 dpi in rats infected with either 500 (Figure 2C) or 10 L3 (Figure 2H). At 40 dpi, the number of circulating leukocytes returned to baseline levels in all dexamethasone-treated rats.

Ivermectin treatment led to a progressive increase in the number of circulating leukocytes, independent of parasite burden (Figure 2A,F). This increase was observed in most cell types (Figure 2), except for circulating eosinophils, which were significantly lower than in rats infected only with the parasite after 20 dpi (Figure 2E,J), suggesting that these cells represent a marker of active infection, as their reduction occurred after parasite elimination.

### 3.3. Reactivity of Different Antibody Isotypes in the Plasma of Rats Experimentally Infected with S. venezuelensis and Treated with Dexamethasone or Ivermectin

**Figure 3:**
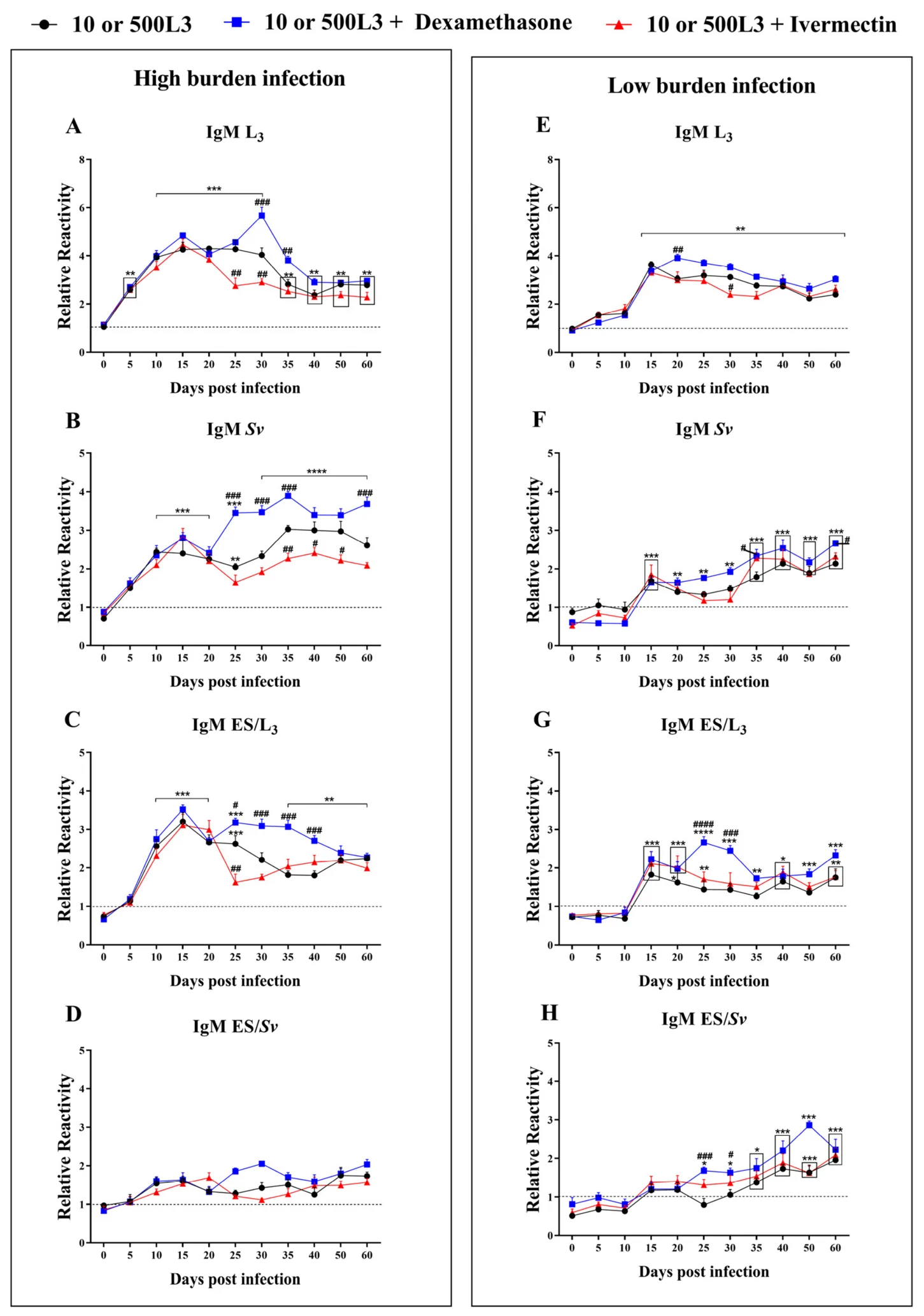
Parasite-specific IgM levels in the plasma of Wistar rats infected with 500 L3 or 10 L3 of *S. venezuelensis*, treated or not treated with dexamethasone or ivermectin. IgM reactivity to L3 (**A**,**E**), adult worm (Sv) (**B**,**F**), excreted–secreted (ES) L3 (**C**,**G**), and ES worm (**D**,**H**) antigens. (**A**–**D**): Rats were infected with 500 L3 per rat (high parasite burden). (**E**–**H**): Rats infected with 10 L3 per rat (low parasite burden). At 0 (before infection), 5, 10, 15, 20, 25, 30, 35, 40, 50, and 60 days after infection, blood was collected from the lateral tail veins to obtain plasma and perform immunoenzymatic assays. 10 L3 or 500 L3 = infected-only animals; 10 L3 + dexamethasone or 500 L3 + dexamethasone = animals infected and treated via IP with 4 mg/Kg/day of Decadron at 15 dpi for 3 days; 10 L3 + ivermectin or 500 L3 + ivermectin = rats infected and treated orally with 200 μg/Kg/day of ivermectin at 15 dpi for 2 consecutive days. Data are plotted as mean ± standard deviation (SD) and were analyzed by means of a two-way mixed-effects model with Geisser–Greenhouse correction, followed by Tukey’s post hoc test. *n* = 7 rats/group. * represents a statistically significant difference in comparison to day 0 or the non-infected control; # represents a statistically significant difference in comparison to the infected-only group at the same time point. # or * *p* < 0.05; ## or ** *p* < 0.01; ### or *** *p* < 0.001; #### or **** *p* < 0.0001. Dashed line represents the cut-off level for antibody reactivity. The boxes highlight diffrent experimental groups that share statistic significance in comparison to unfected (*) or to infected control (#).

In the current study, we evaluated the reactivity of IgM, IgG and the subclasses IgG1 and IgG2a to different parasite antigenic preparations in plasma from infected rats in the different experimental groups to better understand the effects of parasite burden, immunosuppressive treatment, and anthelmintic treatment on the kinetics of antibody production and its association with active infection by *S. venezuelensis*. Plasma IgM reactivity to L3, Sv, and ES/L3 antigens increased in all infected rats, independently of parasite burden; however, low-burden infected rats showed a delayed increase in IgM reactivity (Figure 3E–G) compared with the high-burden infected group (Figure 3A–C). In contrast, IgM reactivity to ES/Sv antigens remained low in the plasma of *S. venezuelensis*-infected rats throughout the experimental period (Figure 3D,H). It is also important to note that dexamethasone treatment did not reduce IgM reactivity, regardless of the antigen extract tested. Moreover, IgM reactivity remained significantly higher in infected rats even after parasite elimination or parasitological cure (Figure 3).

**Figure 4:**
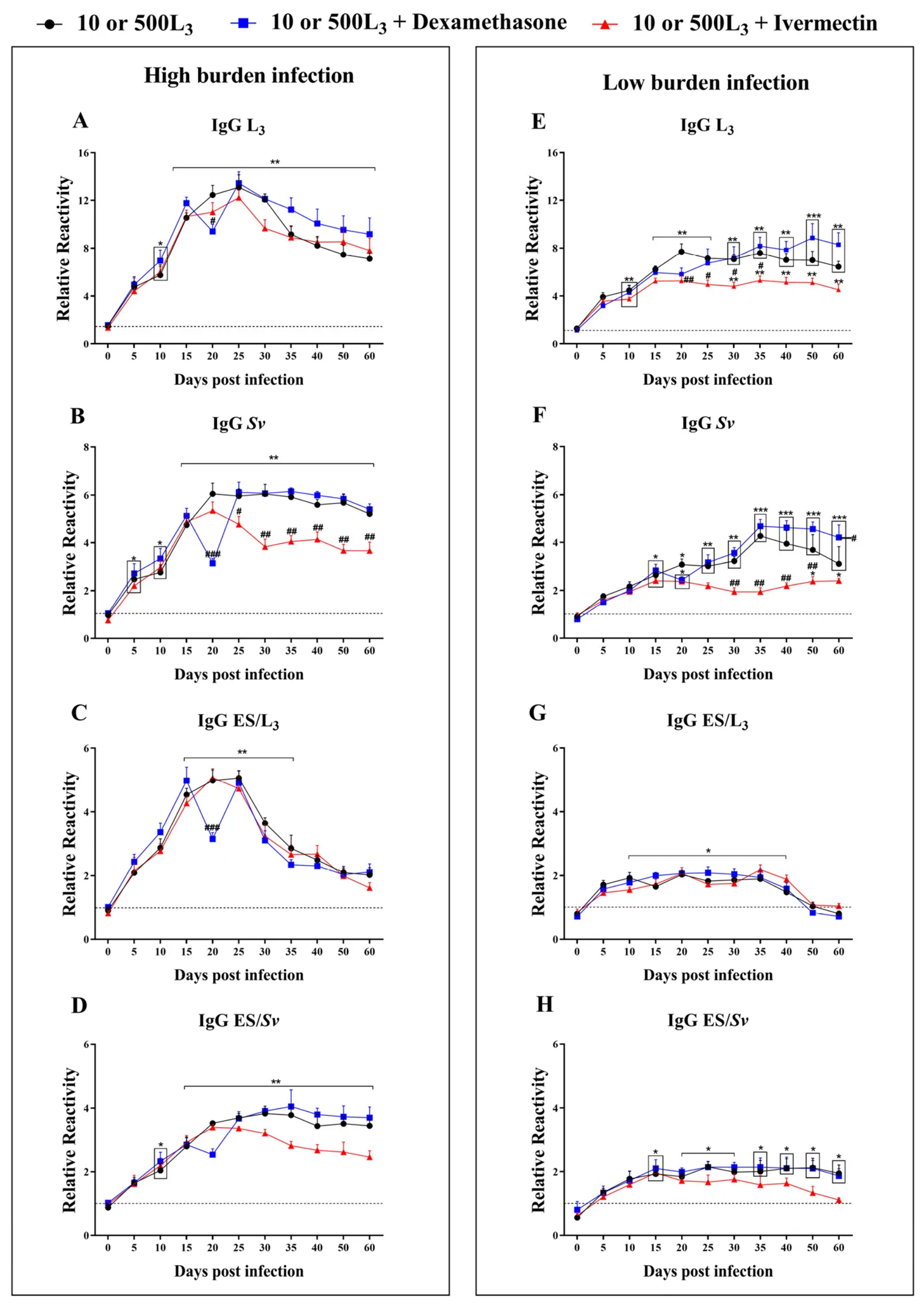
Parasite-specific IgG levels in the plasma of Wistar rats infected with 500 L3 or 10 L3 of *S. venezuelensis*, treated or not treated with dexamethasone or ivermectin. IgG reactivity to L3 (**A**,**E**), adult worm (Sv) (**B**,**F**), excreted–secreted (ES) L3 (**C**,**G**), and ES worm (**D**,**H**) antigens. (**A**–**D**): Rats were infected with 500 L3 per rat (high parasite burden). (**E**–**H**): Rats infected with 10 L3 per rat (low parasite burden). At 0 (before infection), 5, 10, 15, 20, 25, 30, 35, 40, 50, and 60 days after infection, blood samples were collected from the lateral tail veins to obtain plasma and perform immunoenzymatic assays. 10 L3 or 500 L3 = infected-only animals; 10 L3 + dexamethasone or 500 L3 + dexamethasone = animals infected and treated via IP with 4 mg/Kg/day of Decadron at 15dpi for 3 days; 10 L3 + ivermectin or 500 L3 + ivermectin = rats infected and treated orally with 200 μg/Kg/day of ivermectin at 15 dpi for 2 consecutive days. Data are plotted as mean ± standard deviation (SD) and were analyzed by means of a two-way mixed-effects model with Geisser–Greenhouse correction, followed by Tukey’s post hoc test. *n* = 7 rats/group. * represents a statistically significant difference in comparison to day 0 or the non-infected control; # represents a statistically significant difference in comparison to the infected-only group at the same time point. # or * *p* < 0.05; ## or ** *p* < 0.01; ### or *** *p* < 0.001.Dashed line represents the cut-off level for antibody reactivity. The boxes highlight diffrent experimental groups that share statistic significance in comparison to unfected (*) or to infected control (#).

IgG reactivity increased in the plasma of infected rats against all parasite antigens, regardless of parasite burden; however, IgG reactivity against ES antigens was lower in rats infected with a low parasite burden (Figure 4). Moreover, rats infected with a high parasite dose and treated with dexamethasone showed a transient reduction in parasite-reactive IgG levels at 20 dpi compared with the other infected groups; however, parasite-reactive IgG levels increased again after 25 dpi (Figure 4). Plasma IgG reactivity did not return to baseline levels during the course of infection in any of the experimental groups, even after parasite elimination by treatment or spontaneous cure (Figure 4).

**Figure 5:**
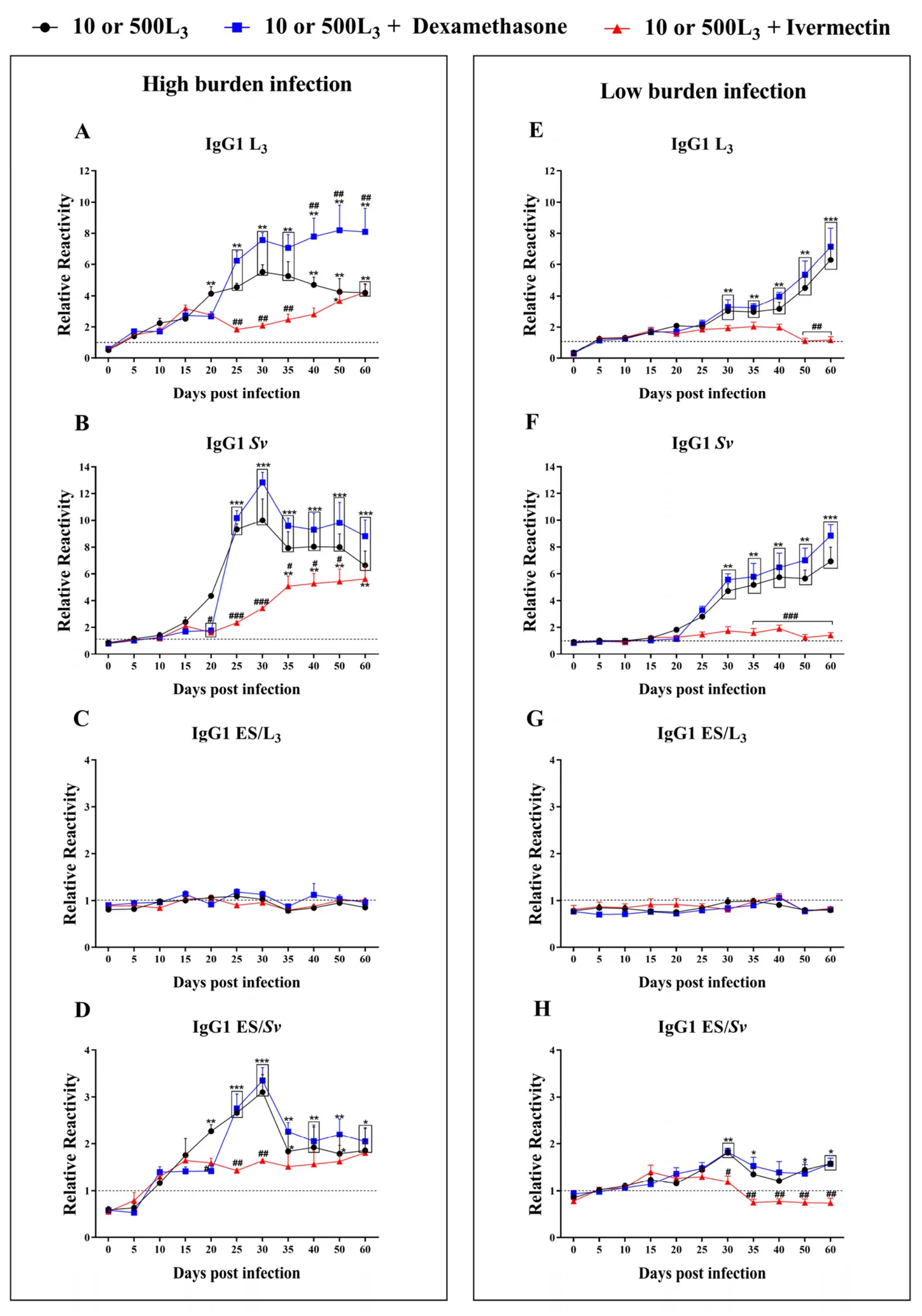
Parasite-specific IgG1 levels in the plasma of Wistar rats infected with 500 L3 or 10 L3 of *S. venezuelensis*, treated or not treated with dexamethasone or ivermectin. IgG1 reactivity to L3 (**A**,**E**), adult worm (Sv) (**B**,**F**), excreted–secreted (ES) L3 (**C**,**G**), and ES worm (**D**,**H**) antigens. (**A**–**D**): Rats were infected with 500 L3 per rat (high parasite burden). (**E**–**H**): Rats infected with 10 L3 per rat (low parasite burden). At 0 (before infection), 5, 10, 15, 20, 25, 30, 35, 40, 50, and 60 days after infection, blood samples were collected from the lateral tail veins to obtain plasma and perform immunoenzymatic assays. 10 L3 or 500 L3 = infected-only animals; 10 L3 + dexamethasone or 500 L3 + dexamethasone = animals infected and treated via IP with 4 mg/Kg/day of Decadron at 15dpi for 3 days; 10 L3 + ivermectin or 500 L3 + ivermectin = rats infected and treated orally with 200 μg/Kg/day of ivermectin at 15 dpi for 2 consecutive days. Data are plotted as mean ± standard deviation (SD) and were analyzed by means of a two-way mixed-effects model with Geisser–Greenhouse correction, followed by Tukey’s post hoc test. *n* = 7 rats/group. * represents a statistically significant difference in comparison to day 0 or the non-infected control; # represents a statistically significant difference in comparison to the infected-only group at the same time point. # or * *p* < 0.05; ## or ** *p* < 0.01; ### or *** *p* < 0.001. Dashed line represents the cut-off level for antibody reactivity. The boxes highlight diffrent experimental groups that share statistic significance in comparison to unfected (*) or to infected control (#).

IgG1 reactivity against L3 (Figure 5A,E), *Sv* (Figure 5B,F), and ES/Sv antigens (Figure 5D,H) significantly increased in the plasma of rats infected with a high parasite burden and in rats infected and treated with dexamethasone only after 20–25 dpi, and it remained elevated even after parasite elimination (Figure 5). Moreover, IgG1 reactivity to parasite antigens was lower in rats infected with a low parasite burden (Figure 5E–H). No detectable IgG1 reactivity to ES/L3 antigens was observed in infected rats from the different experimental groups, regardless of parasite burden (Figure 5C,G). Interestingly, IgG1 reactivity did not increase significantly in rats infected with a low parasite burden and treated with ivermectin at 15 and 16 dpi, whereas the highest increase in plasma IgG1 reactivity in ivermectin-treated rats infected with a high parasite burden occurred after parasite elimination (Figure 5).

**Figure 6:**
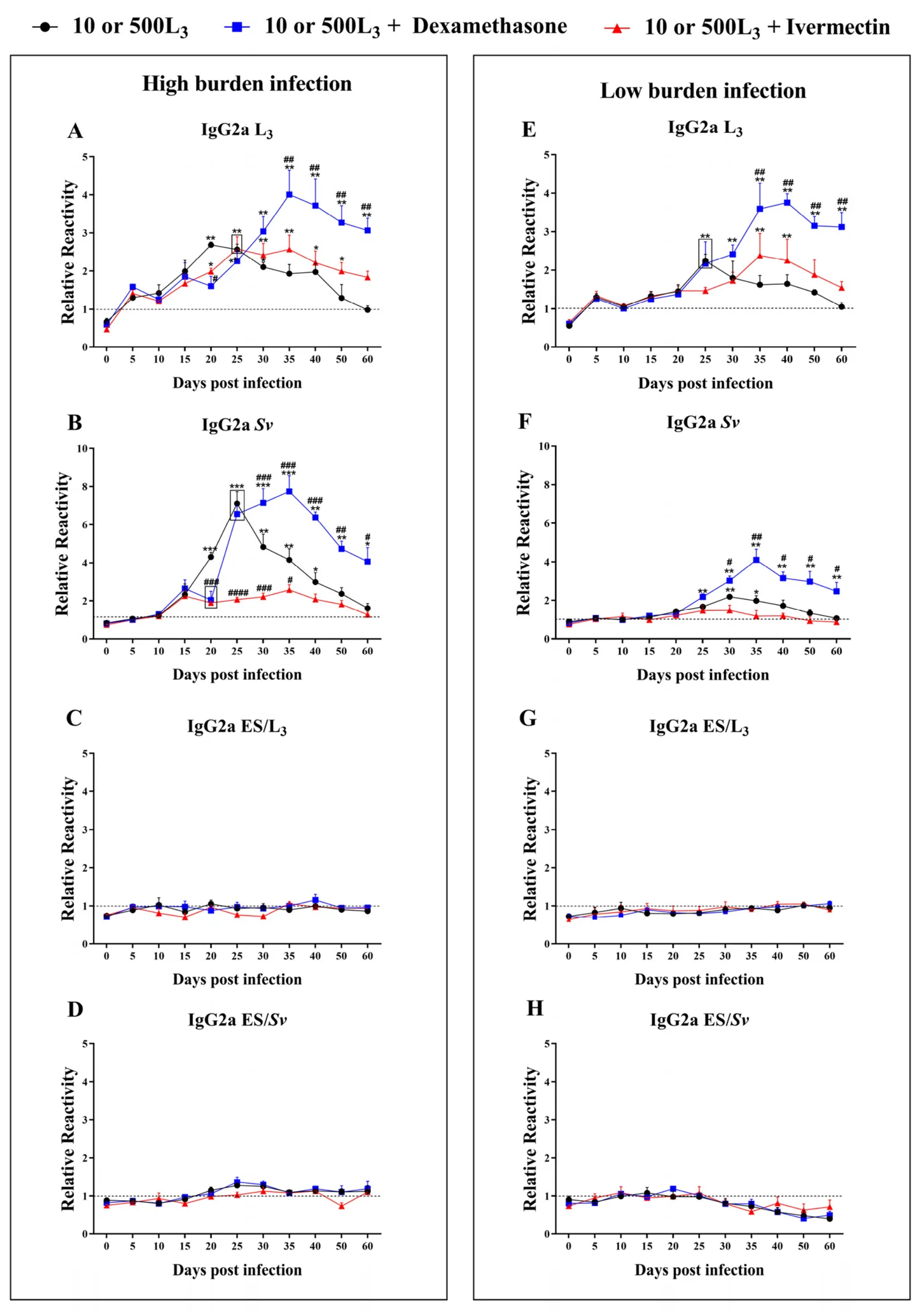
Parasite-specific IgG2a levels in the plasma of Wistar rats infected with 500 L3 or 10 L3 of *S. venezuelensis*, treated or not treated with dexamethasone or ivermectin. IgG2a reactivity to L3 (**A**,**E**), adult worm (Sv) (**B**,**F**), excreted–secreted (ES) L3 (**C**,**G**), and ES worm (**D**,**H**) antigens. (**A**–**D**): Rats were infected with 500 L3 per rat (high parasite burden). (**E**–**H**): Rats infected with 10 L3 per rat (low parasite burden). At 0 (before infection), 5, 10, 15, 20, 25, 30, 35, 40, 50, and 60 days after infection, blood samples were collected from the lateral tail veins to obtain plasma and perform immunoenzymatic assays. 10 L3 or 500 L3 = infected-only animals; 10 L3 + dexamethasone or 500 L3 + dexamethasone = animals infected and treated via IP with 4 mg/Kg/day of Decadron at 15 dpi for 3 days; 10 L3 + ivermectin or 500 L3 + ivermectin = rats infected and treated orally with 200 μg/Kg/day of ivermectin at 15 dpi for 2 consecutive days. Data are plotted as mean ± standard deviation (SD) and were analyzed by means of a two-way mixed-effects model with Geisser–Greenhouse correction, followed by Tukey’s post hoc test. *n* = 7 rats/group. * represents a statistically significant difference in comparison to day 0 or the non-infected control; # represents a statistically significant difference in comparison to the infected-only group at the same time point. # or * *p* < 0.05; ## or ** *p* < 0.01; ### or *** *p* < 0.001; #### < 0.0001. Dashed line represents the cut-off level for antibody reactivity. The boxes highlight diffrent experimental groups that share statistic significance in comparison to unfected (*) or to infected control (#).

All infected groups also showed elevated IgG2a reactivity against L3 (Figure 6A,E) and Sv antigens (Figure 6B,F); however, plasma IgG2a reactivity to ES antigens did not increase in infected rats, independent of parasite burden or treatment (Figure 6C,D,G,H). Moreover, increased IgG2a reactivity to L3 or Sv antigens began after 15 dpi in most infected animals, progressively increased until 30–35 dpi in rats only infected with *S. venezuelensis* or in rats infected and treated with ivermectin. In these experimental groups, IgG2a reactivity showed a progressive reduction after 40 dpi and returned to baseline levels in most animals by 60 dpi (Figure 6A,B,E,F). In contrast, the groups infected and treated with dexamethasone, particularly those infected with a high parasite burden, showed a transient reduction in IgG2a reactivity at 20 dpi, followed by a progressive increase that persisted until the last day evaluated (Figure 6A,B,E,F).

### 3.4. Parasite-Specific IgA Production in Plasma and Intestinal Washes of Rats Experimentally Infected with S. venezuelensis and Treated with Dexamethasone or Ivermectin

In contrast to the IgM and IgG responses, IgA reactivity did not show significant changes in the plasma of infected rats, independent of parasite burden, antigen used in the ELISA, duration of infection, or type of treatment.

**Figure 7:**
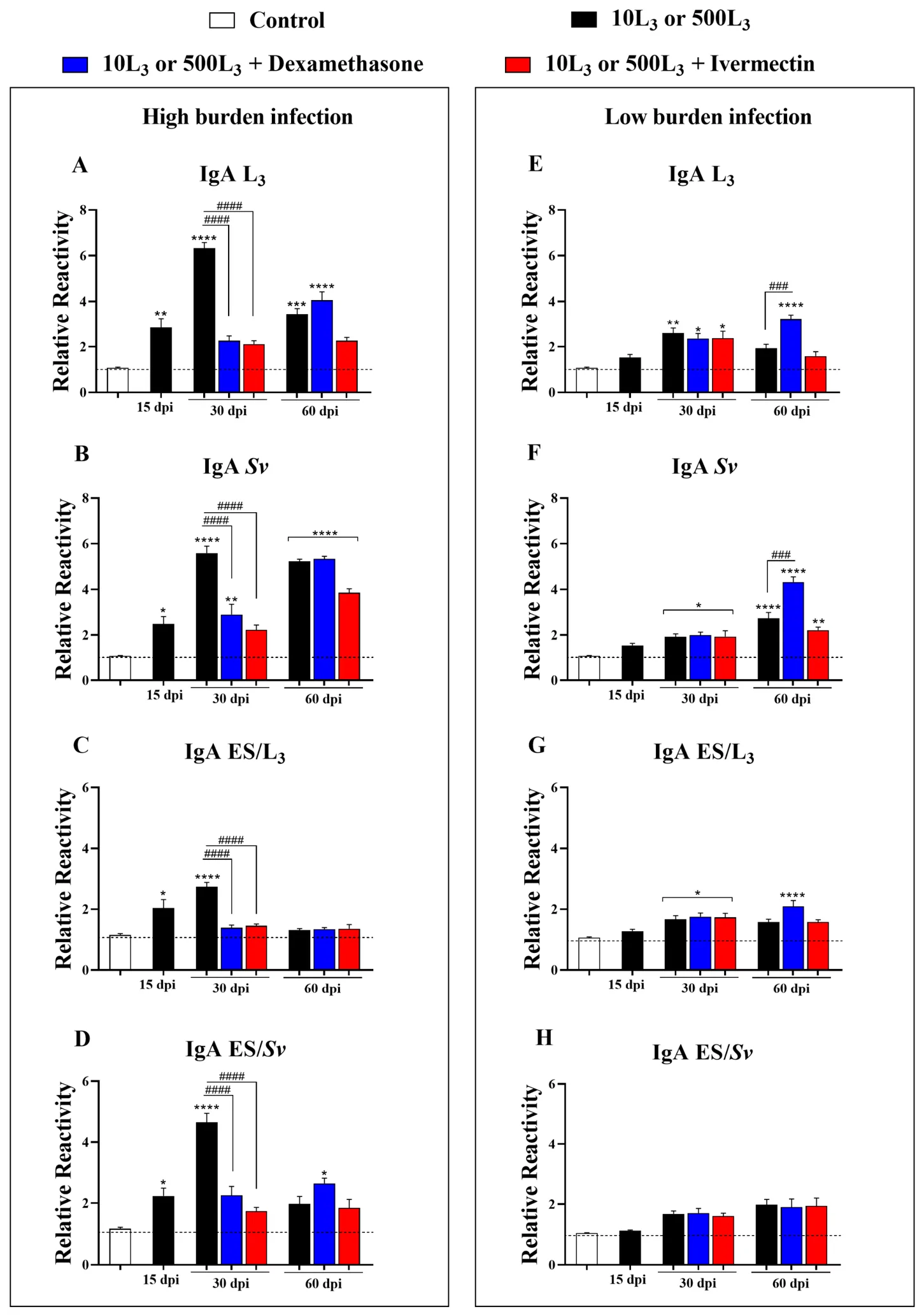
Parasite-specific IgA levels in the intestinal lavage of Wistar rats infected with 500 L3 or 10 L3 of *S. venezuelensis*, treated or not treated with dexamethasone or ivermectin. The IgA reactive to L3 (**A**,**E**), adult worm (**B**,**F**), excreted–secreted (E/S) L3 (**C**,**G**), and ES worm (**D**,**H**) antigens. (**A**–**D**): Rats were infected with 500 L3/rat (high parasite burden). (**E**–**H**): Rats infected with 10 L3/rat (low parasite burden). Intestinal lavage was obtained at 15, 30, and 60 days after infection with the application of 3 mL of sterile PBS containing a protease inhibitor cocktail to the proximal half of the small intestine of experimental animals. The collected material was centrifuged and the supernatant was used in the ELISA assay. Control = uninfected animals; 10 L3 or 500 L3 = infected-only animals; 10 L3 + dexamethasone or 500 L3 + dexamethasone = animals infected and treated via IP with 4 mg/Kg/day of Decadron at 15 dpi for 3 days; 10 L3 + ivermectin or 500 L3 + ivermectin = rats infected and treated orally with 200 μg/Kg/day of ivermectin at 15 dpi for 2 consecutive days. Data are plotted as mean ± standard deviation (SD) and were analyzed by means of two-way ANOVA followed by Tukey’s post hoc test. *n* = 7 rats/group. * represents a statistical difference in comparison to the non-infected control; # represents a statistical difference in comparison to the infected-only group at the same time point. * *p* < 0.05; ** *p* < 0.01; ### or *** *p* < 0.001; #### or **** *p* < 0.0001. Dashed line represents the cut-off level for antibody reactivity.

Although no detectable increase in IgA reactivity was observed in the plasma of infected rats, we evaluated IgA reactivity against different *S. venezuelensis* antigens in the intestinal washes of these animals at three time points during infection. IgA reactivity to L3 and Sv antigens was significantly higher in intestinal washes collected at 15, 30, and 60 dpi, especially in rats infected with 500 L3 per rat. Dexamethasone treatment delayed the detection of reactive IgA in the intestinal wash of infected rats, whereas ivermectin treatment significantly reduced IgA reactivity in intestinal washes (Figure 7A,B,E,F). Notably, IgA reactivity against ES antigens in the intestinal washes of rats infected with a high parasite burden was elevated at 15 and 30 dpi and returned to baseline levels at 60 dpi in rats treated with ivermectin or those that spontaneously eliminated the parasite, suggesting an association with active infection that may be important for diagnostic purposes (Figure 7C,D). However, IgA reactivity to ES antigens was lower in rats infected with a low parasite burden (Figure 7G,H).

## 4. Discussion

Strongyloidiasis is a parasitic infection that remains the most neglected among tropical diseases affecting human populations [6,7,49], mainly due to limitations and challenges associated with diagnostic tests [50,51]. This scenario allows the infection to persist and progress to severe forms of strongyloidiasis. Therefore, more sensitive diagnostic alternatives are needed to identify infections with low parasite burdens and asymptomatic cases [14,52]. However, there are limitations in evaluating such sensitive diagnostic alternatives, especially immunodiagnostic tools, which are difficult to adequately assess in human populations.

In the present study, we evaluated the reactivity of different antibody isotypes against distinct *S. venezuelensis* antigens using Wistar rats as an experimental model. In this model, we demonstrated that circulating eosinophilia, plasma IgG2a reactivity to parasite crude extracts, an antibody isotype associated with type 2 immune responses, and IgA reactivity to L3/ES antigens in intestinal washes were more strongly associated with active infection, even in animals infected with a low parasite burden or treated with ivermectin. However, IgM reactivity to parasite antigens did not decrease in infected animals subjected to immunosuppressive treatment with dexamethasone.

It is important to highlight that in our study, we used rats infected with high and low parasite burdens of *Strongyloides*, which were treated with ivermectin or an immunosuppressant and subjected to periodic parasitological and immunological assessments to confirm their infection status. Although experimental models do not undergo autoinfection and consequently do not develop chronic strongyloidiasis, as observed in human populations, rats are easily maintained under laboratory conditions, have commercially available immunological reagents, and remain infected for a longer period than mice, allowing a more comprehensive evaluation of the humoral response [32,53,54,55]. Moreover, similarly to *S. stercoralis* infection in humans, the infective larvae of *S. venezuelensis* in rats penetrate the skin, migrate through the lungs, and mature into parthenogenetic females within the small intestine [31]. Furthermore, *S. venezuelensis* and *S. stercoralis* share substantial genomic, proteomic, and antigenic homology [5].

Oral administration of ivermectin at 15 and 16 dpi, as applied in the current experimental protocol, resulted in complete elimination of *S. venezuelensis* adult worms from the small intestine, and, consequently, no larvae were detected in the feces of cured rats. The efficacy of ivermectin treatment for *Strongyloides* elimination in rodents has also been demonstrated by Mendonça et al. [56]. In addition, the immunosuppressive effect of the IP administration of dexamethasone, performed between 15 and 17 dpi, was confirmed by a significant reduction in the number of circulating leukocytes across different cell populations in dexamethasone-treated rats compared with all other infected groups. Although the immunosuppressive effect of the protocol used in the current experiment was transient, with a significant reduction in circulating leukocytes occurring from 20 to 35 dpi, the results obtained corroborate the findings in the literature evaluating the effect of dexamethasone in both uninfected rodents [57] and animals infected with *Strongyloides* [58].

In the current study, plasma samples from experimentally infected rats showed an early increase in plasma IgM levels against *Strongyloides* antigens that remained elevated until 60 dpi, independent of parasite burden, spontaneous parasite elimination, ivermectin treatment, or immunosuppressive treatment with dexamethasone. In mice experimentally infected with *Strongyloides* spp., an early increase in IgM reactivity to *Strongyloides* antigens has also been reported, and the production of parasite-reactive IgM has been associated with protective immune responses against migrating larvae [59,60]. Although experimental models do not allow adequate assessment of the chronicity of strongyloidiasis, we observed early detection of anti-*Strongyloides* IgM in the plasma of infected rats, and parasite-specific reactive IgM remained elevated up to 60 days after infection, even in groups infected with a low parasite burden. Moreover, parasite-specific IgM levels remained high during immunosuppressive treatment with dexamethasone in infected rats, regardless of parasite burden. However, it is important to mention that our short-term immunosuppression treatment induced an acute and transient immunosuppression rather than chronic or long-term immunosuppressive conditions typically observed in severe human hyperinfection syndrome. Nevertheless, IgM reactivity persisted and increased immediately after treatment, indicating that IgM responses are not promptly blunted during early immunosuppressive stress. Therefore, our data indicate the resilience of IgM dynamics in the initial phase of immunosuppression and warrant further study in long-term models and in samples of the immunosuppressed human population to better evaluate their use as an immunodiagnostic alternative to confirm strongyloidiasis in immunosuppressed patients.

Although the kinetics of IgM anti-*Strongyloides* responses are poorly documented in human populations, some studies have reported early elevation of reactive serum IgM in individuals with acute strongyloidiasis [3,61,62]. Additionally, some studies suggest that IgM reactivity may be used for diagnostic screening in individuals suspected of *S. stercoralis* infection, especially in non-endemic areas, as IgM levels are markedly elevated at the beginning of infection and may remain detectable for extended periods [9]. However, the present data also demonstrated that plasma IgM reactivity in infected rats did not allow differentiation between active infection with a low parasite burden and treated or cured animals. This potential limitation of a diagnostic approach based on parasite-specific IgM levels should be further investigated in human populations in endemic areas.

In the current study, we also evaluated the reactivity of total IgG and its isotypes, IgG1 and IgG2a, to different *S. venezuelensis* antigenic preparations, as IgG is the main immunoglobulin class present in serum, representing 70–75% of total serum antibodies [9]. Similarly to the pattern observed for reactive IgM, IgG reactivity to *Strongyloides* antigens increased in rats from all infected groups, independent of parasite burden. However, a significant and transient reduction in IgG reactivity was observed after dexamethasone treatment at 20 dpi, and plasma parasite-reactive IgG levels did not return to baseline after parasite elimination, a finding that limits their use for diagnostic purposes.

Regarding the transient reduction in IgG levels observed in rats from the groups infected and treated with dexamethasone for three days after 15 dpi, this effect may be explained by inhibition or lysis of B lymphocytes, which are highly sensitive to glucocorticoids [63], thereby directly affecting IgG production on a temporary basis. A study conducted in rats infected with *S. venezuelensis* and immunosuppressed for 30 days with dexamethasone reported IgG levels below the detection limit at all evaluated time points [64]; these data corroborate our findings at 20 dpi. It should be emphasized that although daily administration ensures continuous immunosuppression, the mechanical stress to which the animals are subjected in this treatment protocol represents a disadvantage that may compromise animal welfare and increase susceptibility to opportunistic infections [58]. Thus, although the immunosuppressive treatment used in this study transiently suppressed aspects of the humoral response, the data suggest that IgG reactivity may not be a reliable marker of active parasite infection.

Indeed, experimental infections with *S. venezuelensis* in mice [65] and rats [64] have demonstrated elevated levels of specific IgG, and this increase persists even after self-cure and/or parasite elimination. Previous studies conducted in patients infected with *S. stercoralis* have also shown that IgG reactivity increases after the sixth week of infection and remains elevated for prolonged periods, even after treatment [21,66,67,68], indicating that IgG reactivity is not a reliable marker of active *Strongyloides* infection in experimental models or in human populations. Moreover, although commercial tests and kits based on total IgG are available for *Strongyloides* diagnosis, parameters associated with the duration of IgG reactivity following parasitological cure, the occurrence of cross-reactivity with other parasitic helminths, and efficacy in immunosuppressed individuals remain poorly evaluated in human populations, especially in endemic areas [9,13,69,70].

Published data [5,9,67] support the hypothesis that the reactivity of different antibody isotypes could improve the accuracy of serological diagnosis for strongyloidiasis. Experimental strongyloidiasis in rats may also help clarify the association between active infection and IgG isotype reactivity levels. In the current study, we evaluated IgG1 and IgG2a reactivity in infected rats subjected to different treatments. Our data demonstrated that a significant increase in IgG1 reactivity to parasite crude extracts occurred later after infection, especially in animals with a low parasite burden, and did not decrease after spontaneous parasite elimination, again indicating limitations for diagnostic purposes. Moreover, IgG1 reactivity to parasite ES antigens was very low and did not allow differentiation between infected and uninfected control animals, regardless of parasite burden. On the other hand, IgG2a isotype reactivity showed potential to differentiate untreated infected rats from ivermectin-treated rats that achieved parasitological cure, including animals with a low parasite burden. In addition, IgG2a reactivity was less susceptible to the immunosuppressed treatment used in the experimental procedure, particularly a few days post-treatment, when parasite burden was also high. However, in the immunosuppressed group, IgG2a reactivity remained elevated even after spontaneous parasitological clearance. Altogether, these findings demonstrate that IgG2a serves as a reliable marker to confirm clearance in ivermectin-treated groups and remains resilient following acute immunosuppression. However, its diagnostic value in immunosuppressed contexts is limited by the persistence of elevated IgG2a reactivity even after active parasite clearance. Previous studies have shown that rats infected with *Strongyloides* spp. had elevated IgG2a levels compared with uninfected animals [64,71]. However, these studies did not evaluate the diagnostic performance of this antibody isotype, providing only data related to immune responses during *Strongyloides* infection or to the effects of immunosuppression on immunological and parasitological parameters in infected rats [71]. In humans, IgG4 is frequently associated with IL-4 production and Th2 response [72], leading some studies to propose a functional analogy with rat IgG2a [73]. However, such cross-species functional parallels remain a subject of discussion, and subclass profiles alone should be interpreted with caution in the absence of direct cytokine or cellular polarization markers. As we observed for IgG2a in rats experimentally infected with *S. venezuelensis*, several investigations have shown that IgG4 levels are elevated in individuals with active *S. stercoralis* infection compared with treated and cured individuals [74,75,76]. However, only a few studies have specifically evaluated the diagnostic performance of IgG4 reactivity in human strongyloidiasis, showing promising results [77,78]. As previously mentioned, although direct cross-species functional homology between rat IgG2a and human IgG subclasses (such as IgG4) is not fully supported by the literature [73], the reactivity of IgG2a anti-L3 or adult worm antigens in our assay serves as a robust proof-of-concept. It demonstrates that the target antigen possesses high immunogenicity and exposes epitopes suitable for high-affinity antibody binding. Monoclonal or specific rat IgG2a antibodies with high affinity for *Strongyloides* antigens can be directly exploited in competitive ELISAs or sandwich-type immunoassays to capture circulating parasite antigens or outcompete non-specific human antibodies, thereby improving diagnostic specificity. However, it should be noted that our findings may not directly apply in immunosuppressed contexts of *Strongyloides* infection; therefore, further validation is required, particularly using human samples from patients with diverse health conditions.

Regarding IgA reactivity to different *Strongyloides* antigens, the present data demonstrated low IgA reactivity in the plasma of infected rats across the different experimental groups; however, high IgA reactivity was detected in intestinal lavage samples from infected rats, regardless of parasite burden. The presence of parasite-reactive IgA in intestinal lavage samples, but not in plasma, is consistent with the *Strongyloides* spp. life cycle, in which larvae migrate through the lungs and become established within the small intestinal mucosa, thereby inducing local IgA production [79,80]. Furthermore, following parasitological cure, we demonstrated that anti-ES/L3 IgA levels returned to baseline values, mainly in high burden groups. IgA is the predominant immunoglobulin class in mucosal secretions, with peak production occurring at the beginning of the second week of *Strongyloides* infection and remaining elevated until the fifth week [9,81]. Data from the literature indicate that the presence of IgA in mucosal secretion samples may reflect active infection or possible reinfection, and the negative anti-*Strongyloides* IgA results or a progressive reduction in IgA levels may indicate parasite elimination [24,82,83]. Although the detection of IgA in intestinal lavage provides direct proof-of-concept for local mucosal immune activation against *Strongyloides*, its diagnostic application in humans is limited by the invasive nature of the sampling method. To translate these mucosal findings to clinical practice, testing more accessible, non-invasive biological samples, such as stool or saliva samples, represents a logical next step. However, the diagnostic utility of mucosal IgA remains unproven because its reactivity to parasite antigens was low in lightly infected animals, which represent the most difficult cases to diagnose in human populations.

In addition to parasite-specific antibody production, *Strongyloides* spp. infection also alters other parameters of the host immune response, such as circulating leukocyte counts in peripheral blood, which may contribute to the diagnosis of strongyloidiasis. Our data showed that although different leukocyte populations increased in the peripheral blood of infected rats, only eosinophil counts decreased following ivermectin treatment and spontaneous parasitological clearance, particularly in high-burden groups. However, eosinophil counts also declined in animals with active infection treated with dexamethasone, which may represent a diagnostic limitation for this specific subgroup in human populations. Traditionally, eosinophils have been considered important effector cells involved in protection against certain helminth infections [84]. Specifically, in strongyloidiasis, eosinophilia is frequent and may be long-lasting, even in asymptomatic cases [85]. From a clinical perspective, increased eosinophil counts in peripheral blood represent a nonspecific finding, as various helminth infections and allergic diseases can induce eosinophilia [86,87]. However, some published studies have associated blood eosinophilia with strongyloidiasis, particularly in travelers, immigrants, and refugees who visited or lived in endemic areas [88,89]. It is important to note that in most of these studies, parasitological examinations were not performed, which precludes definitive attribution of eosinophilia to *S. stercoralis* infection. Therefore, our data suggest that eosinophil counts may serve as a complementary parameter to support the identification of active *Strongyloides* spp. infection.

It is important to note that the lack of non-infected control groups treated solely with dexamethasone or ivermectin represents a limitation of our study design. Although this limits our ability to entirely isolate direct drug effects from infection-induced immune responses, the comparison between infected non-treated and infected treated groups successfully captured key shifts in parasite-specific antibody production under immunosuppression and anthelmintic treatment.

## 5. Conclusions

In summary, the present study demonstrates time-dependent immunological patterns temporally associated with experimental infection and parasite elimination, particularly plasma IgG2a reactivity to somatic extracts and intestinal IgA reactivity, even in low parasite burden scenarios. In contrast, total IgM reactivity exhibited high resilience across all experimental conditions, highlighting its potential relevance during early acute immunosuppressive stress. Furthermore, circulating eosinophil counts proved to be a dynamic complementary marker that closely reflects infection status and therapeutic resolution. Although these experimental findings require formal diagnostic accuracy testing and validation in human clinical samples, this controlled model provides essential proof-of-concept evidence and candidate immunological targets to support the rational design of improved stage-specific diagnostic alternatives for human strongyloidiasis.

## Figures and Tables

**Table 1:** Reagents and dilutions used in the ELISA test applied to plasma samples of rats uninfected and infected with a low or high burden of *S. venezuelensis*, treated or not with immunosuppressant or anthelmintic.

Assay	Blocking Solution	Antibody Specification	Working Dilution
IgM	1% BSA	Goat Polyclonal Anti-Rat IgM—Conjugate Biotin (Bethyl Laboratories Inc., Montgomery, TX, USA)	1:10,000
IgG	1% BSA	Goat Polyclonal Anti-Rat IgG-Fc Fragment—Conjugate Biotin, Bethyl Laboratories Inc.	1:5000
IgA	1% BSA	Goat Polyclonal Anti-Rat IgA—Conjugate Biotin, Bethyl Laboratories Inc.	1:5000
IgG1	2% skimmed milk	Goat Polyclonal Anti-Rat IgG1—Conjugate HRP, Bethyl Laboratories Inc.	1:3000
IgG2a	2% skimmed milk	Goat Polyclonal Anti-Rat IgG2a—Conjugate HRP, Bethyl Laboratories Inc.	1:3000

**Table 2:** The average number of worms per intestine and larvae per gram of feces counted in Wistar rats infected with different burdens of *S. venezuelensis* and treated or not treated with dexamethasone or ivermectin, after 15, 30, and 45 days of infection.

High Parasite Burden						
DPI	500 L3		500 L3 + Dexamethasone		500 L3 + Ivermectin	
	Adult Worms ^a^	Larvae ^b^	Adult Worms ^a^	Larvae ^b^	Adult Worms ^a^	Larvae ^b^
15	78.1 ± 7.7	3806.1 ± 553.3	78.1 ± 7.7	3689.0 ± 469.9	78.1 ± 7.7	3581.3 ± 744.5
30	36.3 ± 2.8	356.2 ± 176.0	57.3 ± 5.6 *	824.5 ± 529.5 *	0	0
45	0	0	0	0	0	0
**Low parasite burden**						
**DPI**	**10 L3**		**10 L3 + Dexamethasone**		**10 L3 + Ivermectin**	
	**Adult worms ^a^**	**Larvae ^b^**	**Adult worms ^a^**	**Larvae ^b^**	**Adult worms ^a^**	**Larvae ^b^**
15	3.4 ± 0.4	615.1 ± 107.6	3.4 ± 0.4	671.4 ± 89.3	3.4 ± 0.4	640.2 ± 133.7
30	1.1 ± 0.3	254.0 ± 132.4	1.7 ± 0.2	451.9 ± 97.3 **	0	0
45	0	0	0	0	0	0

DPI = days post-infection; ^a^ average number of worms per intestine; ^b^ average number of larvae per g of feces intestine; 10 L3 or 500 L3 = number of infective larvae (10 or 500) used for infection; 10 L3 + dexamethasone or 500 L3 + dexamethasone = the infective dose used in each animal (10 or 500) followed by treatment with 4 mg/Kg/day of Decadron, via IP, at 15 dpi for 3 days; 10 L3 + ivermectin or 500 L3 + ivermectin = the infective dose used in each animal (10 or 500) followed by treatment with 200 μg/Kg/day of ivermectin, orally, at 15 and 16 dpi. The values at 15 dpi were obtained before treatment. Data are presented as mean ± standard deviation (SD) and were analyzed using the Kruskal–Wallis test followed by Dunn’s post hoc correction. *n* = 7 rats/group. * represents a statistically significant difference in comparison to the infected-only group at the same time point. * *p* < 0.05; ** *p* < 0.01.

## Data Availability

Data are contained within the article.
